# Atypical Fracture of the Scapular Spine: A Case Report

**DOI:** 10.7759/cureus.60237

**Published:** 2024-05-13

**Authors:** Ryosuke Mashiko, Michiyuki Hakozaki, Yoichi Kaneuchi, Takuya Nikaido, Yoshihiro Matsumoto

**Affiliations:** 1 Department of Orthopaedic Surgery, Fukushima Prefectural Minami-Aizu Hospital, Fukushima, JPN; 2 Department of Orthopaedic Surgery, Fukushima Medical University School of Medicine, Fukushima, JPN; 3 Higashi-Shirakawa Orthopaedic Academy, Fukushima Medical University School of Medicine, Fukushima, JPN

**Keywords:** elderly patient, round back, bone-modifying agent, bisphosphonate, scapular fracture, atypical fracture

## Abstract

Atypical fractures are gaining attention as a severe potential side effect of long-term treatment with bone-modifying agents (e.g., bisphosphonate and denosumab) for osteoporosis. Most atypical fractures occur in weight-bearing bones; the femur is the most frequent site. Atypical fractures occurring in non-weight-bearing bones are extremely rare. We describe an atypical fracture of the scapular spine in a 92-year-old Japanese woman with osteoporosis who had been treated with minodronate for ~7 years. Although the dislocation of the fracture site remained after conservative treatment, there was no obstacle to her daily life.

## Introduction

With the background of the globally aging population and the high awareness of osteoporosis and osteoporosis-related fragility fractures [1], the number of individuals diagnosed with osteoporosis and requiring medical treatment is increasing. Bone-modifying agents (BMAs), including bisphosphonate and denosumab, are widely used to treat osteoporosis, but some severe potential side effects of long-term BMA treatment have emerged: osteonecrosis of the jaw and atypical fractures [1-4]. Most of the reported atypical fractures occurred in weight-bearing bones, and the femur is the most frequently affected weight-bearing bone (atypical femoral fracture; AFF) [2]. Although the absolute risk of AFF in patients treated with bisphosphonates is reported to be between 3.2 and 50 cases per 100,000 person-years [5], reports of atypical fractures that occurred in a non-weight-bearing bone are extremely rare, especially in the scapula, and there are no reports of their incidence. We describe the case of an atypical fracture of the scapular spine of an elderly woman who had received long-term bisphosphonate treatment.

## Case presentation

**Figure 1:**
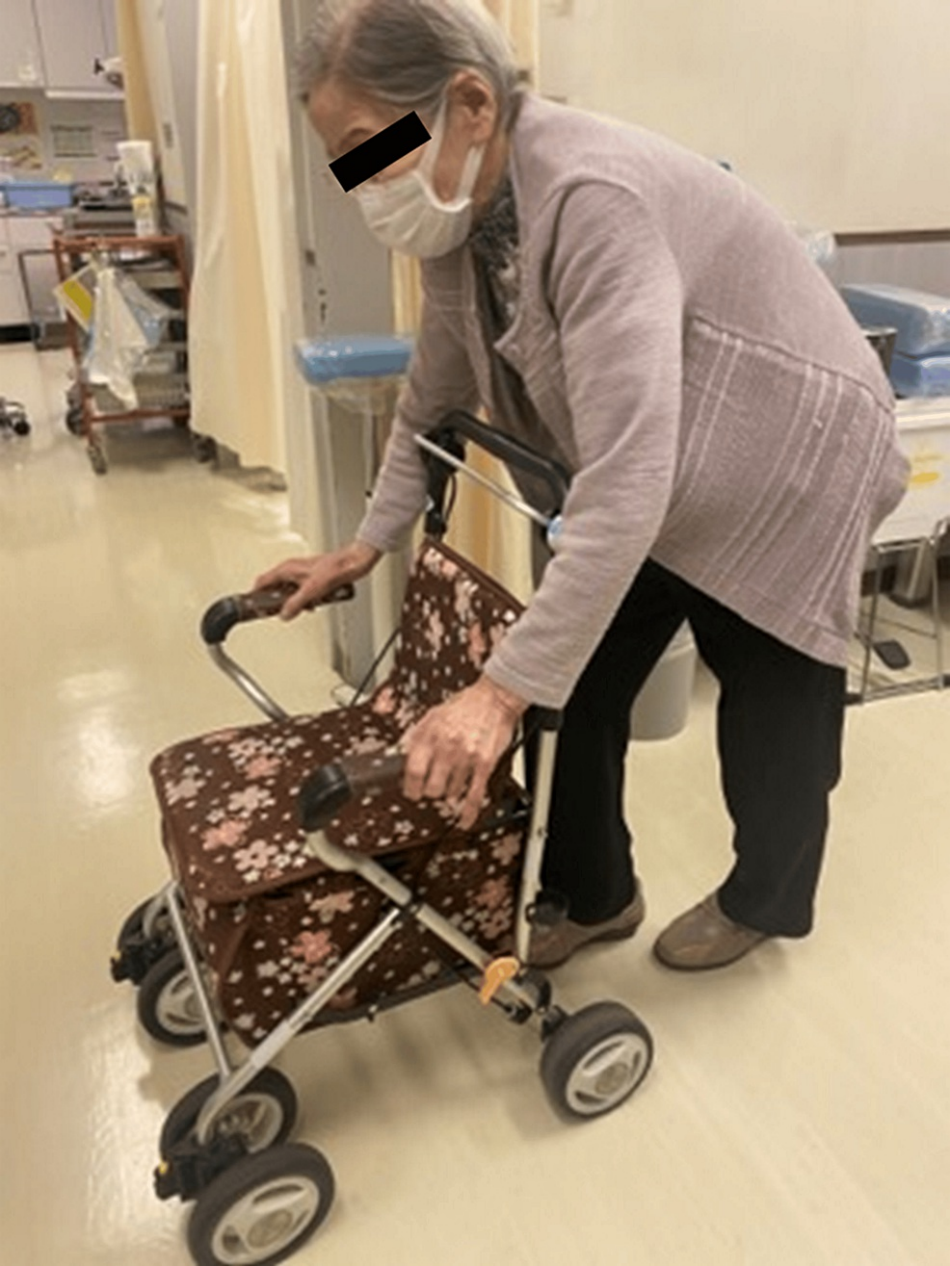
Photograph of the patient She walked with a wheeled walker because of her round back.

The patient was a 92-year-old Japanese woman with a history of two times of thoracolumbar compression fractures at T12 and L4. She was diagnosed with osteoporosis and had been treated with 50 mg per month of minodronate for approximately 7 years. As she was classified as a high-risk individual according to the National Osteoporosis Guideline Group update 2013 in the UK [6] (age over 75 years and history of vertebral fractures), her treatment with minodronate was continued without a withdrawal period. She was right-handed and usually walked with a wheeled walker because of her round back (Figure 1).

**Figure 2:**
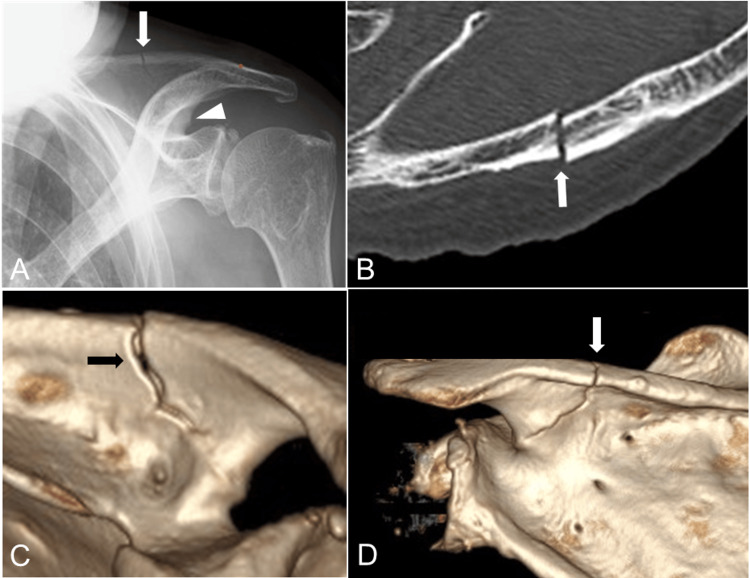
Radiological findings at the patient's initial visit Plain radiography (A) and CT (B, axial view; C, anteroposterior view of 3D-CT; D, posteroanterior view of 3D-CT) shows a transverse fracture (arrows) of the scapular spine, but the alignment is almost normal, and a beak was formed at the scapular notch (arrowhead).

When she pulled her pants on with both hands one day, she experienced severe pain in her left shoulder and simultaneously heard the sound of bone breaking, and she was unable to lift her pants further. She thus came to our hospital, and on examination, mild tenderness and swelling in the left scapular spine area were observed but subcutaneous hemorrhage was not observed. Although her left shoulder motion was painful, she was able to elevate and abduct her shoulder >90°. Plain radiographs and computed tomography showed a transverse fracture of the scapular spine, but the alignment was almost normal, and a beak was formed at the scapular notch (Figure 2).

**Figure 3:**
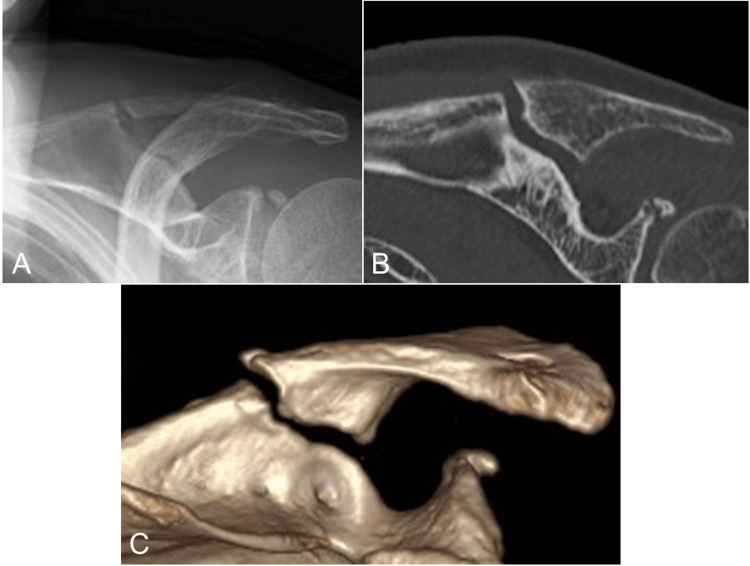
Radiological findings at 15 months after the injury Plain radiography (A) and CT (B, coronal view and C, anteroposterior view of 3D-CT) show the remaining dislocation of the fracture.

Although Levy's classification [7] is used for periprosthetic fractures that have undergone a reverse shoulder arthroplasty, if this classification is applied mutatis mutandis (i.e., the necessary changes have been made) to the present fracture, it would be classified as Type 2. Since the patient had been taking bisphosphonate for a long time before this injury, her case fulfilled all five of the major features of the 2010 AFF criteria issued by the American Society for Bone and Mineral Research [5], and a diagnosis of atypical scapular spine fracture was made. Imaging analyses of the contralateral scapula, bilateral femurs, lower legs, and forearms were also performed, but there were no obvious beak signs or strong bowing. Considering the patient's advanced age, her bisphosphonate regimen was stopped, and she was treated conservatively with subcutaneous administration of teriparatide (20 μg per day) for approximately 9 months and oral administration of eldecalcitol (0.75 μg per day). Dislocation of the fracture progressed gradually, but the patient's pain was under control, and she declined surgical treatment. Fifteen months after the injury, the dislocation of the fracture remained (Figure 3), but there was no obstacle to her activities of daily living (ADLs).

## Discussion

The risk of AFF due to long-term BMA treatment is becoming widely recognized, and the number of patients diagnosed with AFF is increasing. Severely suppressed bone turnover by long-term BMA treatment [8] and repeated loading stress associated with a bowing deformity of the femoral shaft [9] are the factors reported as causes of AFF. The most frequent site of atypical fractures of a long bone is the femur, followed by the tibia, both of which are weight-bearing bones; however, atypical fractures can occur in various bones [10]. Atypical fractures in an upper limb are rare, but several cases of an atypical ulnar fracture (AUF) are reported [11-14]. Only two cases of atypical scapular fractures have been reported: one in the acromion [15] and the other in the scapular spine [16]. The reported risks of AUF, which is a non-weight-bearing part of the body, include forward-leaning posture due to a round back and the load on the proximal forearm caused by daily work with the elbows on a table [11,12]. In our patient's case, in addition to the long-term use of bisphosphonate, she had been using her left, non-dominant upper limb for support when holding the standing posture, which resulted in chronic stress on her left shoulder and an atypical fracture of the scapular spine.

Along with suppressed bone turnover, AFFs are known to be prone to nonunion. Because the femur is a weight-bearing bone and its nonunion may highly reduce a patient's ADLs, an early discontinuance of BMA treatment and the introduction of another drug such as teriparatide after surgical treatment are mandatory for the faster healing of AFFs [3,17]. Moreover, Tsuchie et al. reported that the administration of teriparatide after the occurrence of the first AFF and the use of active vitamin D3 after the completion of teriparatide therapy may prevent the recurrence of AFF [18]. Concerning the non-weight-bearing bones, Abe et al. reviewed the reported cases of AUFs and described the difficulty in the treatment of AUFs as has been encountered with AFFs [13]. In the cases of the two reported patients and the present patient with atypical scapular fracture, BMA treatment was discontinued and the patients were treated conservatively. The patient with the atypical scapular fracture in the acromion achieved bone union after 1 year [15], but in the case of the atypical scapular fracture in the scapular spine, the final result was not stated [16]. Our patient's fracture showed nonunion 15 months after her presentation. Although the risk of nonunion in atypical scapular fractures is considered high, the scapular spine is a non-weight-bearing bone, and the indication for surgery should be determined based on the patient's intentions and the degree of ADL impairment.

## Conclusions

We have reported an extremely rare case of an atypical fracture of the scapular spine of an elderly woman who had received long-term bisphosphonate treatment. Atypical fractures can occur even in non-weight-bearing bones, and the scapular spine should be recognized as a risk area in addition to the ulna in the upper limb. The risk of nonunion in such cases is expected to be as high as that observed for the femur, and the indications for surgery should be thoroughly discussed with the patient.
